# Towards an advanced therapy medicinal product based on mesenchymal stromal cells isolated from the umbilical cord tissue: quality and safety data

**DOI:** 10.1186/scrt398

**Published:** 2014-01-17

**Authors:** José Paulo Martins, Jorge Miguel Santos, Joana Marto de Almeida, Mariana Alves Filipe, Mariana Vargas Teixeira de Almeida, Sílvia Cristina Paiva Almeida, Ana Água-Doce, Alexandre Varela, Mari Gilljam, Birgitta Stellan, Susanne Pohl, Kurt Dittmar, Werner Lindenmaier, Evren Alici, Luís Graça, Pedro Estilita Cruz, Helder Joaquim Cruz, Rita Nogueira Bárcia

**Affiliations:** 1 ECBio, Investigação e Desenvolvimento em Biotecnologia, S.A., Amadora, Portugal; 2 Instituto de Medicina Molecular, Faculdade de Medicina da Universidade de Lisboa, Lisbon, Portugal; 3 Cell and Gene Therapy Centre, Department of Medicine, Division of Hematology, Karolinska Institutet, Karolinska University Hospital, Huddinge, Sweden; 4 Helmholtz-Centre for Infection Research, Department of Gene Regulation and Differentiation, Braunschweig, Germany

## Abstract

**Introduction:**

Standardization of mesenchymal stromal cells (MSCs) manufacturing is urgently needed to enable translational activities and ultimately facilitate comparison of clinical trial results. In this work we describe the adaptation of a proprietary method for isolation of a specific umbilical cord tissue-derived population of MSCs, herein designated by its registered trademark as UCX®, towards the production of an advanced therapy medicinal product (ATMP).

**Methods:**

The adaptation focused on different stages of production, from cell isolation steps to cell culturing and cryopreservation. The origin and quality of materials and reagents were considered and steps for avoiding microbiological and endotoxin contamination of the final cell product were implemented. Cell isolation efficiency, MSCs surface markers and genetic profiles, originating from the use of different medium supplements, were compared. The ATMP-compliant UCX® product was also cryopreserved avoiding the use of dimethyl sulfoxide, an added benefit for the use of these cells as an ATMP. Cells were analyzed for expansion capacity and longevity. The final cell product was further characterized by flow cytometry, differentiation potential, and tested for contaminants at various passages. Finally, genetic stability and immune properties were also analyzed.

**Results:**

The isolation efficiency of UCX® was not affected by the introduction of clinical grade enzymes. Furthermore, isolation efficiencies and phenotype analyses revealed advantages in the use of human serum in cell culture as opposed to human platelet lysate. Initial decontamination of the tissue followed by the use of mycoplasma- and endotoxin-free materials and reagents in cell isolation and subsequent culture, enabled the removal of antibiotics during cell expansion. UCX®-ATMP maintained a significant expansion potential of 2.5 population doublings per week up to passage 15 (P15). They were also efficiently cryopreserved in a DMSO-free cryoprotectant medium with approximately 100% recovery and 98% viability post-thaw. Additionally, UCX®-ATMP were genetically stable upon expansion (up to P15) and maintained their immunomodulatory properties.

**Conclusions:**

We have successfully adapted a method to consistently isolate, expand and cryopreserve a well-characterized population of human umbilical cord tissue-derived MSCs (UCX®), in order to obtain a cell product that is compliant with cell therapy. Here, we present quality and safety data that support the use of the UCX® as an ATMP, according to existing international guidelines.

## Introduction

The public clinical trials database [1] currently shows approximately 130 open clinical trials using mesenchymal stromal cells (MSCs) for a very wide range of therapeutic applications, the majority of which are in Phase I (safety studies), Phase II (efficacy studies) or combined Phase I/II studies. Clinical trials using MSCs are showing promising results. This has resulted in an increase in demand for the development of production processes in accordance with guidelines for Good Manufacturing Practices (GMP), to guarantee reliability of the cells for the purpose of their use in clinical studies and ultimately, the advancement of stem cell-based therapies (for an extensive review, see [2]).

Due to the novelty, complexity and technical specificity of cell therapy, specially tailored and harmonized regulations were necessary to ensure global availability of cellular products. Currently, in the European Union, the regulation (EC) No. 1394/2007 on Advanced Therapy Medicinal Products (ATMPs) lays down specific guidelines concerning centralized authorization, supervision and pharmacovigilance.

One of the most important requirements of ATMPs is the full characterization of the product. Safety is a major concern with this type of biopharmaceutical. The cell-based product must not cause infections, allergies or malignancies. To verify this, numerous quality control steps need to be implemented within the manufacturing process, including microbiological testing (to detect viral, fungal, mycoplasma or contamination with other bacteria) and pyrogenicity testing. In addition, a phenotype analysis must also be performed in order to assess identity and the degree of purity of the cell population as well as additional safety testing, including genetic stability and tumorigenicity (even if human MSCs are thought not to transform *in vivo*) [3,4].

In addition to safety, cell number is another major concern in cell-based products since a significant number of functional cells is needed for clinically relevant cell dosages. Recent clinical trials have used minimum multiple dosages of 1 to 2 × 10^6^ cells/kg of body weight for graft-versus-host disease in transplant patients [5], myocardial infarction [6] and in the treatment of Crohn’s disease [7].

The umbilical cord is a rich source of MSCs which, unlike other sources, does not require invasive harvesting procedures. Because of their multi/pluripotency, immunomodulatory and homing properties, as well as their capacity to produce valuable cytokines and/or growth factors, these cells are interesting candidates for therapeutic applications in immune-related disorders and regenerative medicine [8,9].

We developed and patented an isolation method that consistently isolates MSCs from the umbilical cord tissue that meet the International Society for Cellular Therapy (ISCT) guideline criteria [10]. The cell-based product was registered under the UCX® trademark. UCX® isolated using this patented method have proven to be able to induce immune tolerance *in vivo* by repressing T-cell activation and promoting the expansion of Tregs, and in a chronic adjuvant induced arthritis model, animals treated with UCX® showed faster remission of local and systemic arthritic manifestations [9].

In the present work, we adapted our proprietary method for the production of UCX® so they can be certified as an ATMP, both for autologous and allogeneic, off-the-shelf, cell therapy applications. The adaptation occurred at different stages of production, from cell isolation steps to cell culturing and cryopreservation. The cell product that resulted from the selected method was finally termed UCX®-ATMP, and was characterized in terms of cell identity, purity (microbiological, identity and viability), tumorigenicity and genetic stability. Some general potency assays were also performed confirming the potential of the UCX®-ATMP product to become an ATMP.

## Materials and methods

### Umbilical cord samples

This study was performed in accordance with the Declaration of Helsinki and approved by the Ethics Committee at the Cascais Hospital Dr. José de Almeida. Umbilical cord donations were obtained with written informed consents according to Directive 2004/23/EC of the European Parliament (Portuguese Law 22/2007 of June 29).

### Isolation of UCX® and UCX®-ATMP

Human umbilical cord tissue MSCs were isolated according to a proprietary method [10]. The resulting cell product is termed UCX®. The ATMP adaptation of this method focused on different stages of manufacturing: 1) an initial decontamination step was introduced, using an efficient antibiotic/antimycotic solution in order to avoid the use of antibiotics and antimycotics in the cell culture medium during the expansion step; 2) clinical grade enzymes were used and fetal bovine serum (FBS) substitutes were introduced to improve safety of the cellular product; and 3) sample handling materials and the nature of cell culture reagents were altered to ensure a mycoplasma- and endotoxin-free cellular product.

For the isolation of UCX®, umbilical cord sections, from either vaginal or caesarean delivery, were transported to the laboratory facilities and processed within a period up to 72 hours after collection. The umbilical cords were immersed in a decontaminating solution overnight at 4°C prior to processing. The cords were washed and the outer amniotic membrane removed. Transversal sections of 2.5 cm were then digested with a mixture of either: 1) standard crude collagenase, type II (Sigma-Aldrich), and porcine trypsin (Sigma-Aldrich), the herein named enzyme research grade method (ENZ(RG)), or 2) GMP grade equivalent collagenase and animal component free TrypLE™ Select (Gibco®, Life Technologies™, Carlsbad, CA, USA), the herein named enzyme clinical grade method (ENZ(CG)).

An optimized precise proportion among tissue mass, enzyme activity units, digestion solution volume and void volume was used for the isolation of cells from the umbilical cord tissue. A total of 17.5 g of umbilical cord was processed per isolation (on average, 1 cm corresponds to 1 g of UC tissue). Cells from both the first and second recovery phases (as described in Santos *et al*., 2006 [10]) were joined together and used as a single culture. After digestion, static horizontal incubation and centrifugation, the cell suspension was incubated in static monolayer culture flasks (Nunc, Thermo Fisher Scientific, Roskilde, Denmark) at 37°C in a humidified atmosphere containing 7% CO_2_ in basal MSC culture medium (α-MEM basal medium with 1 g/L glucose and 2 mM glutamine (Sigma-Aldrich®, St. Louis, MO, USA)) supplemented with either 20% FBS (Invitrogen™, Life Technologies™, Carlsbad, California, USA), 20% human serum (HS) (Lonza, Walkersville, MD, USA) or 20% human Platelet Lysate (hPL) (provided by the ^©^Karolinska Institutet, Stockholm, Sweden, SE).

Platelet lysate was prepared in-house from five to six buffycoats (containing SSP + additive solution, Macopharma, Tourcing, France) from A + donors and plasma. Human serum (Lonza) was obtained from human AB blood from normal human donors who tested negative for hepatitis B surface antigen (HbsAG), antibodies to hepatitis C (HCV), and human immunodeficiency virus I and II (HIV-I and HIV-II). Sera were pooled and sterile filtered.

After overnight incubation, non-adherent cells were removed and fresh medium was added. Cultures were maintained and a complete change of culture medium was performed twice weekly. Fibroblast-like colonies were observed regularly and passaged when 80 to 90% confluence was observed.

Cells were detached using either 0.25% (w/v) Trypsin-EDTA (Sigma-Aldrich), in the ENZ(RG) protocols, or with TrypLE™ Select (Gibco) in the ENZ(CG) protocols. MSC medium was added and cells counted and seeded at 5,000 to 10,000 cells/cm^2^ on culture flasks (Nunc). Cells were placed at 37°C, 7% CO_2_ in a humidified incubator, and fed by replacing the culture medium twice weekly until 80 to 90% confluence.

For the ATMP-adapted protocols, all autoclaved glassware material was substituted with plastic ware and all sample handling and cell culture materials were certified to be mycoplasma-free and certified as having an endotoxin level <0.1 EU/mL.

Cultures were monitored on a regular basis for the visible detection of bacterial or fungal contaminations. Near confluent cell culture supernatants were collected at different passages and analyzed for mycoplasma and endotoxin contamination using the LookOut® Mycoplasma PCR Detection Kit (Sigma-Aldrich) and the Limulus Amebocyte Lysate (LAL) PYROGENT® Plus (Lonza) respectively, according to the manufacturer’s instructions.

### Expression profiling

Total RNA from UCX® cultivated in MSC-medium supplemented with FBS, HS or hPL was isolated using the RNeasy Mini Kit (QIAGEN, Valencia, CA, USA) following the protocols of the manufacturer. About 5 × 10^6^ cells were collected after trypsinization. After cell lysis, homogenization was performed by passing the lysate five times through a 20-gauge syringe and DNAse digestion was used to eliminate DNA contamination. Quality and integrity of the total RNA isolated was controlled on a bioanalyzer (Agilent Technologies, Santa Clara, CA, USA).

Total RNA, weighting 5 μg, was used for biotinylated target synthesis according to standard protocols supplied by the manufacturer (Affymetrix, Santa Clara, CA, USA). Briefly, RNA was converted to dsDNA using 100 pmol of a T7T23V primer (Eurogentec, Seraing, Belgium) containing a T7 promoter. The cDNA was then used directly in an *in vitro* transcription reaction in the presence of biotinylated nucleotides. The concentration of biotin-labeled cRNA was determined by UV absorbance. For hybridization, 10 μg of each biotinylated cRNA preparation were fragmented and placed in a hybridization cocktail containing also four biotinylated hybridization controls (BioB) as recommended by the manufacturer. Samples were hybridized for 16 hours to Affymetrix Gene Chip HG_U133 Plus 2.0, representing about 47,000 human transcripts. After hybridization the GeneChips were washed, stained with SA-PE and read using an Affymetrix GeneChip fluidic station and scanner.

The resulting dataset is available under Gene Expression Omnibus (GEO) accession number GSE51869. Analysis of microarray data was performed using the Affymetrix Microarray Suite 5.0 and BRB Array Tools 4.2. All array experiments were normalized using Robust Multi-array Average. Correlation of expression profiles from differentially cultivated UCX® cells was done using BRB Array Tools and visualized as scatter plots.

### Immune phenotypic analyses

To analyze cell-surface expression of typical MSC surface marker proteins, cells were detached, counted and labeled with the following anti-human antibody conjugates: CD44 - APC; CD73 - APC; CD90 - PE; CD14 - PerCp/Cy5.5; CD45 - PerCp/Cy5.5; CD31 - FITC; CD34 - FITC; CD19 - Pacific Blue; HLA-DR - Pacific Blue (all from Biolegend) and also CD105 PE (eBioscience, Inc., San Diego, CA, USA). The mouse isotype antibodies used as the respective controls were: Pacific Blue IgG1; Pacific Blue IgG2a; IgG1k PerCp/Cy5.5; IgG2a PerCp/Cy5.5; IgG1k PE; IgG1k APC and IgG1k FITC (all from Biolegend®, San Diego, CA, USA). A total of 10,000 labeled cells were acquired using a Gallios Flow cytometer (Beckman Coulter) and results analyzed with Kaluza software (Beckman Coulter, Inc., Carlsbad, CA, USA).

### Cryopreservation of UCX® and UCX®-ATMP

Approximately 3 × 10^6^ cells were centrifuged at 200 *g*, at 4°C for 10 minutes and UCX® were resuspended in 1 mL of 90% (v/v) FBS (Invitrogen) with 10% (v/v) DMSO (Sigma-Aldrich) and UCX®-ATMP in Biofreeze (Biochrom, Merck Millipore, Berlin, Germany). Cells were cryopreserved using a Controlled Rate Freezer (CRF) (IceCube14S, Sylab, Neupurkersdorf, Austria) and a freezing profile adapted from Freimark in 2011 [11].

### Thawing of UCX® and UCX®-ATMP

Cryovials were removed from the liquid N_2_ container and placed immediately in the water bath at 37°C until cell suspension was almost entirely thawed. Cell suspension was then diluted one-tenth with pre-warmed MSC medium, counted and plated in a culture flask (Nunc) at approximately 20,000 cells/cm^2^ and placed in a humidified incubator at 37°C, 7% CO_2_.

### Differentiation of UCX®-ATMP

Adipogenic differentiation was induced by cyclic changes of induction and maintenance media in cells cultivated post-confluence as previously described [9]. After three cycles of media changes, adipogenic differentiation was apparent by intracellular accumulation of lipid-rich vacuoles that stained with Oil Red O.

To promote chondrogenic differentiation, cell pellets were prepared and cultured for three weeks in complete chondrogenic differentiation medium, as previously described [9]. After the culture period, fixed, deparaffinized and rehydrated sections were stained with 1% (w/v) alcian blue (Sigma-Aldrich) in 3% (v/v) acetic acid (Sigma-Aldrich) and bright blue stained glycosaminoglycans and mucopolysaccharides were visible.

Osteogenic induction medium was used to promote differentiation as previously described [9]. The onset of osteoblast formation was evaluated after four weeks by the detection of alkaline phosphatase activity using the leukocyte alkaline phosphatase-kit (Sigma-Aldrich) according to the manufacture’s protocol.

### MSC karyotyping and evaluation of teratoma-forming potential

Freshly thawed UCX®-ATMP cells were cultured overnight using the above mentioned culture conditions with the addition of colchicine (Sigma-Aldrich) at a final concentration of 5 μg/mL. Cells were then washed and resuspended with hypotonic (0.56%) potassium chloride solution for 10 minutes and then fixed in acetic acid:methanol at a 1:3 ratio. Slides were then flame-dried to improve the spread of the chromosomes.

The conventional Q-banding technique was used according to the International System for Human Cytogeneic Nomenclature, ISCN 2005, and cytogeneic analysis was performed with a Cytovision Image analysis system (Applied Imaging, Santa Clara, CA, USA). A total of 20 metaphases were analyzed. Two cells with identical structural abnormalities, two with identical supernumerary chromosomes or three with the same missing chromosomes were considered to constitute an abnormal clone.

The teratoma formation assays with UCX®-ATMP were conducted in the Animal Centre of the Karolinska Institutet (SE), a licensed animal research facility and was approved by the ethics committee of South Stockholm under the number S166/01. A total of 12 immunodeficient male, C.B.-17/GbmsTac-scid-bgDF N7 mice (six weeks old), were obtained from M&B, Ltd., Ry (Denmark) and kept under isolated conditions in M2 cages on aspen wood chips (Beekay bedding, Scanbur B&K AB, Karlslunde, Denmark) with free access to water and rodent diet, using artificial light from 6:00 to 18:00, Room temperature: 24 ± 2°C, and humidity: 55 ± 10%. Briefly, cells were thawed 16 hours prior to implantation and approximately 1 × 10^4^ cells were implanted beneath the testicular capsule of a young (six-week) SCID/Beige male mouse anesthetized using isoflurane (Baxter, Deerfield, IL, USA). Human embryonic stem cell line H-9 was used as a positive control. Teratoma growth was determined by palpation and the mice were sacrificed (cervical dislocation) 6.5 to 8.5 weeks post implantation. The tissue was fixed in 4% neutral buffered formaldehyde overnight, dehydrated through a graded series of alcohols to xylene, and embedded in paraffin.

### Mixed lymphocyte reaction

Peripheral blood samples were obtained from healthy volunteers of both genders after informed consent. Blood was collected, diluted 1:1 (v/v) with PBS (1×) and mixed with half the volume of Histopaque®-1077 (Sigma-Aldrich). Peripheral blood mononucleated cells (PBMCs) were collected from the Ficoll gradient after centrifugation at 720 g for 30 minutes at RT.

The Mixed Lymphocyte Reaction (MLR) was performed in 96-well microtiter plates using RPMI (Gibco) and 5% HS obtained from the specific donor. The PBMCs from two different donors were cultured at 2 ×10^5^ cells per well. Stimulator cells (UCX® or UCX®-ATMP) were irradiated with 50 Gy (Gammacell ELAN 3000, Best Theratronics, Ltd., Ottawa, Ontario, Canada) prior to addition to the culture, at 20,000 cells per well. Quadruplicate cultures were performed for each condition. Cultures were incubated at 37°C in 5% CO_2_ for six days, pulsed with [3H]thymidine (1 microCi per well, Amersham Biosciences, Piscataway, NJ, USA) for 16 hours, and the cells were harvested onto filter mats using a Tomtec 96-well cell harvester (Perkin Elmer). Radioactivity incorporated into the dividing cells was determined using a scintillation counter (Microbeta Trilux Scintillation and Luminescence Counter 145 LSC, Perkin Elmer, Waltham, MA, USA).

### Immune suppression assay

The immune suppression assay was performed in 96-well microtiter plates using RPMI (Gibco) supplemented with 5% HEPES (Gibco), 5% Pen-Strep (Gibco), 5% NaPyr (Gibco) and 5% human serum obtained from the specific donor. The PBMCs were obtained from two different donors and cultured at 2 × 10^5^ cells per well and were stimulated with anti-CD3 (eBioscience), anti-CD28 (eBioscience) and IL-2 (eBioscience). Suppressor cells (UCX® or UCX®-ATMP), were irradiated with 50 Gy (Gammacell ELAN 3000, Best Theratronics) prior to addition to the culture at 50,000 cells per well. Quadruplicate cultures were performed for each condition. Cultures were incubated at 37°C in 5% CO_2_ for six days, pulsed with [3H]thymidine (1 microCi per well, Amersham Biosciences, Piscataway) for 16 hours, and the cells were harvested onto filter mats using a Tomtec 96-well cell harvester (Perkin Elmer). Radioactivity incorporated into the dividing cells was determined using a scintillation counter (Microbeta Trilux Scintillation and Luminescence Counter 145 LSC, Perkin Elmer).

### Treg conversion assay

PBMCs were collected and washed with PBS containing 2% FCS and then stained with mAbs against human CD3, CD4 and CD25 (eBioscience) for cell sorting. The purified CD3^+^CD4^+^CD25^-^ T-cells were cultured in plate-bound αhuCD3 (2.5 μg/mL, eBioscience) in 96-well flat-bottom plates in the following conditions. Briefly, 1 × 10^5^ purified T-cells/well were cultured in the presence of 2 μg/mL αhuCD28 (eBioscience), 20 U/mL huIL-2 (Peprotech®, Rocky Hill, NJ, USA) and 10 ng/mL TGF-β (R&D Systems®, Minneapolis, MN, USA) or the indicated UCX® (irradiated as described), in replacement of TGF-β, in a ratio of 1:1 to the T-cells. All conditions were performed in triplicate wells. After five days in culture at 37°C with 5% CO_2_, cells were stained with mAbs against human CD3, CD4 and CD25 (eBioscience) and then stained for huFoxp3 as described by the manufacturer (eBioscience). The analysis was performed on the converted CD3^+^CD4^+^CD25^+^Foxp3^+^ regulatory T-cells.

## Results

### Evaluation of candidate UCX®-ATMP isolation protocols

ECBio has previously developed a reproducible technology for the isolation, culture and cryopreservation of a specific population of umbilical cord tissue-derived MSCs [10]. This cell product was characterized previously according to the ISCT guidelines [12] and the resulting product recently registered with the UCX® trademark. In this study, various steps of the original manufacturing process were adapted to be in conformity with European Medicines Agency (EMA)’s quality and safety guidelines in order to allow for the clinical use of the UCX® cell product as an ATMP. Namely, clinical grade (CG) enzymes were incorporated to replace research grade (RG) enzymes in the digestion process and the isolated cells cultured in FBS substitutes, such as hPL and HS. Sample handling and cell culture materials and solutions were also altered in order to deter possible microbiological and pyrogenic contaminations. For the purpose of this work, the original manufacturing process is referred to as ENZ(RG) FBS, the manufacturing process involving clinical grade enzymes and human serum is referred to as ENZ(CG) HS, and the manufacturing process involving clinical grade enzymes and human platelet lysate is referred to as ENZ (CG) hPL (see the Materials and methods section).

A pre-evaluation of the storage/decontamination time and temperature was performed by a paired analysis of UCs (n = 5). Results showed that, for a successful decontamination, an overnight storage at 4°C is more effective (100% decontamination) than incubation for one hour at 37°C (80% decontamination). Both storage/decontamination methods used did not cause any significant alterations in the average number of cells collected at P0 or in the necessary time to reach confluence. However, alterations in cell yield occurred when cords were stored for over 72 hours.

**Figure 1:**
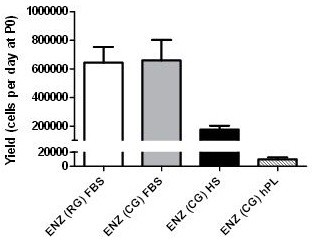
**Isolation of UCX® cells.** A standard protocol using a mixture of crude collagenase and porcine trypsin for cell isolation (ENZ(RG)), and fetal bovine serum (FBS) for cell culture was adapted by replacing the digestion enzymes with clinical grade enzymes (ENZ(CG)) and culturing the cells in human serum (HS) or human Platelet Lysate (hPL). The efficiency of the process was plotted taking into consideration the total cells at the end of passage 0 (P0) isolated from a standard cord (17.5 g, considering an average weight of 1 gram per centimeter of cord), and the days to confluence. Results are represented as mean ± SEM.

The effects of changing the digestion enzymes and serum supplements were also evaluated. For that purpose, the yields of the different isolation methods were analyzed and compared. The digestion of the umbilical cords using RG and CG reagents, followed by culture of isolated cells in FBS provided similar cell yields, indicating that the introduction of CG enzymes alone does not affect the method’s isolation efficiency (Figure 1). In contrast, cell isolation and subsequent culture in the presence of the FBS substitutes, HS or hPL, showed a significant decrease (*P* <0.01) in cell yields, namely, a 4-fold reduction with HS and a dramatic 68-fold decrease with hPL (Figure 1). This result suggests that the adherence and/or start-up growth of UCX® cells are affected by the FBS substitutes. For hPL cultures, there were visibly less adherent cells on the day after isolation in comparison with the cells cultured in HS. Since so few cells were scattered throughout the culture surface, they formed confluent clusters and not a homogeneous monolayer and consequently, cells took significantly longer to reach local confluence at the end of passage 0 (P0), that is, to undergo the initial selection phase of adherence and then grow and multiply. Interestingly, when cells from three different cords isolated with FBS containing media were thawed at different passage numbers (p5, p10 e p15) and cultured in hPL supplemented media, proliferation was slightly higher in hPL-containing cultures when compared to FBS (see Additional file 1: Figure S1). We, therefore, speculate that the observed reduced isolation efficiency in hPL-supplemented cultures is associated with poor initial cell adherence and not with proliferation.

**Figure 2:**
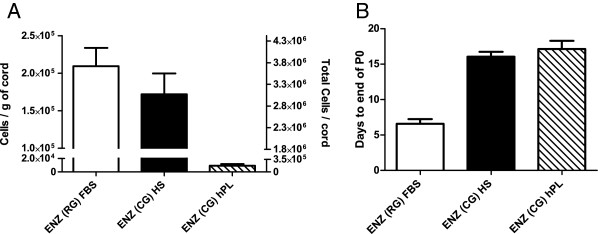
**Cell isolation yields. (A)** Cell isolation yield in cells/g of cord (left y axis) and in cells per cord (17.5 g) (right y axis) at the end of passage 0 (P0) and **(B)** time necessary for the culture to reach confluence. Results are represented as mean ± SEM.

**Figure 3:**
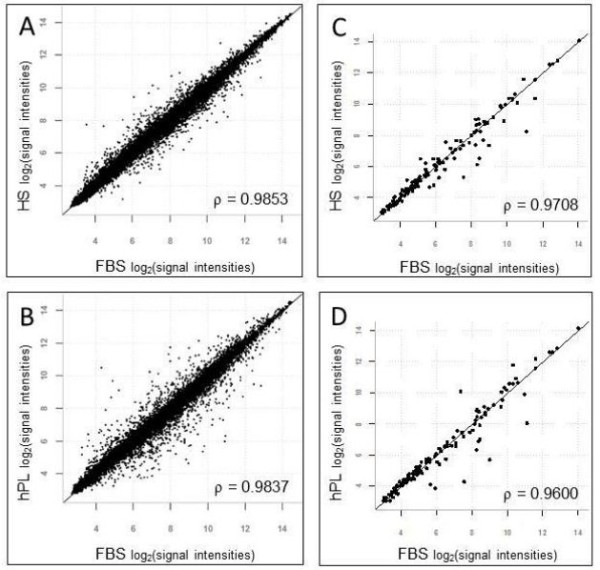
**Gene expression profiles of UCX® cultured in HS (A, C) and hPL (B, C) versus FBS.** Scatterplots for all genes represented on the HG-U133Plus2.0 array **(A, B)** or 132 selected MSC-relevant gene sets representing surface markers and immune suppression related genes **(C, D)** are shown. Mean Spearman pairwise correlation coefficients (ρ) are indicated. FBS, fetal bovine serum; hPL, human Platelet Lysate; HS, human serum.

In HS cultures, an extended number of days was required for the cultures to reach confluence at P0 (17 days, Figure 2), suggesting that UCX® had longer duplication times in HS than in FBS. An Affymetrix gene expression array (HG U133 Plus 2.0, representing >47,000 transcripts) was used to compare the gene expression profiles of the cell populations obtained when cells are cultured in the FBS substitutes. Results showed that the expression profiles of the cells from both HS and hPL cultures were generally similar (ρ >0.96) in comparison to those from the ENZ(RG) FBS protocol (Figure 3A, B). However, when cultures in hPL were compared to cultures in FBS (from the same cord), 1,103 probe sets (683 genes) were found to be regulated more than two-fold. A total of 488 genes were down-regulated and according to Database for Annotation, Visualization and Integrated Discovery (DAVID)-Functional Annotation Clustering these are mostly enriched for “tube formation-lung development, cell migration and differentiation”. Of the genes that were up-regulated (195 genes), most were enriched for “extracellular protein and adhesion” matches.

When cultures in HS were compared to cultures in FBS (from the same cord) the signals from 803 probe sets (corresponding to 517 genes) were altered more than two-fold. A total of 372 gene transcripts were down-regulated and 195 gene transcripts were up-regulated. According to DAVID-Functional Annotation Clustering, the down-regulated genes were mostly enriched for “cell cycle, regulation, transcription”, potentially connected to slower growth, whereas the up-regulated genes were mostly enriched for matches within the “extracellular matrix” functional annotation group.

A narrower analysis of the expression signals resulting from probe sets for MSC relevant genes involved in immune regulation, antigen presentation, growth control and MSC markers also revealed a more prominent gene expression down-regulation effect in UCX® grown in hPL than in HS (Figure 3C, D). Table 1 summarizes the genes with more than two-fold altered expression in both FBS substitutes. In this cluster, we observed that the expression of seven genes was altered when FBS was substituted by HS, while the use of hPL resulted in double the number of genes with altered expression.

**Table 1:** Gene expression in cells cultured with HS or hPL as substitutes of FBS

**Gene name**	**Symbol**	**>two-fold change**	**>two-fold change**
		**HS-FBS**	**hPL-FBS**
chemokine (C-C motif) ligand 5	*CCL5*		↓
fibroblast growth factor 2 (basic)	*FGF2*		↓
fibroblast growth factor 7	*FGF7*	↓	↓
hepatocyte growth factor (hepapoietin A; scatter factor)	*HGF*	↓	↓
insulin-like growth factor binding protein 5	*IGFBP5*	↓	
interleukin 1, beta	*IL1B*	↓	↓
interleukin 6 (interferon, beta 2)	*IL6*	↓	↓
keratinocyte growth factor-like protein 2	*KGFLP2*		↓
v-kit Hardy-Zuckerman 4 feline sarcoma viral oncogene homolog	*KIT*		↓
prostaglandin E synthase	*PTGES*		↓
fibroblast growth factor 5	*FGF5*		↑
latent transforming growth factor beta binding protein 2	*LTBP2*		↑
5′-nucleotidase, ecto (CD73)	*NT5E*	↓	
platelet derived growth factor D	*PDGFD*	↑	
platelet-derived growth factor receptor, alpha polypeptide	*PDGFRA*		↑
prostaglandin E receptor 4 (subtype EP4)	*PTGER4*		↑
transforming growth factor, beta receptor II (70/80 kDa)	*TGFBR2*		↑

FBS, fetal bovine serum; hPL, human Platelet Lysate; HS, human serum.

163 Affymetrix-clustered genes described to be involved in immune regulation, antigen presentation, growth control, and MSC markers were analyzed.

The hPL-specific down-regulation of certain genes, such as *CCL5*, *FGF2* and *PTGES* (which regulates the expression of PGE2), could partially compromise the potential therapeutic effects attributed to MSCs through paracrine recruitment of resident MSCs, angiogenesis and immunosuppression, respectively [13-17]. If we consider FBS cultures as a reference (with proven therapeutic effects) [9], these results indicate that a greater similarity was observed when cells are cultured in HS.

**Figure 4:**
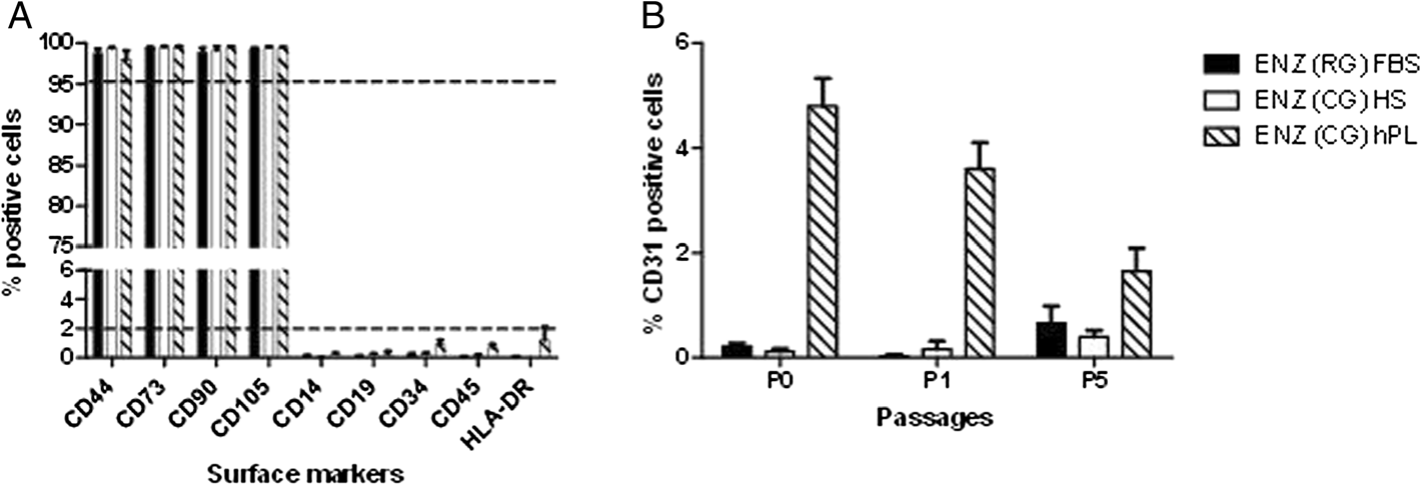
**Flow cytometric analysis of cell surface markers in cell populations isolated and cultured in FBS, hPL and HS (at the end of passage 0).** Comparison of **(A)** ISCT minimal MSC markers, including CD44 and **(B)** the CD31 endothelial marker from passage 0 through to passage 5, from the various protocols. Results are represented as mean ± SEM. FBS, fetal bovine serum; hPL, human Platelet Lysate; HS, human serum; ISCT, International Society for Cellular Therapy; MSC, mesenchymal stromal cell.

Flow cytometric analysis, performed at the end of P0, demonstrated that cells from the ENZ(CG) HS, ENZ(CG) hPL protocols and ENZ(RG) FBS cultured cells, all express the surface marker profile defined by the ISCT for MSCs (>95% positive for the surface markers CD105, CD90 and CD73 and <2% for CD45, CD34, CD14, CD19 and HLA-DR). Additionally, CD44, a mediator protein of cell-to-cell interactions, cell adhesion and migration, generally associated with MSC phenotype was also highly expressed in all UCX® cell populations analyzed. hPL-cultured cells did, however, show a slight but significant increase in HLA-DR expression; although cells remained <2% positive for this surface marker (Figure 4A). More notably, the ENZ(CG) hPL-derived cell population showed an increased number of cells expressing CD31, a platelet endothelial cell adhesion molecule. Although not an ISCT MSC requisite, it is generally accepted that MSCs are devoid of this endothelial marker [18,19]. CD31 positive cells were detected in ENZ(CG) hPL-derived cells at P0, P1 and P5, though showing a markedly decreasing trend in CD31 expression with each cell passage, and reaching levels below 2% by P5 (Figure 4B).

The hPL-induced changes observed in surface marker expression corroborated the gene expression profiling in the sense that UCX® undergo greater gene expression deviations from the original ENZ(CG) FBS population when cultured in medium with hPL in comparison to HS.

Such divergence could have a negative impact on UCX® therapeutic value. Thus, due to higher phenotypic similarities and yields with the ENZ(RG) FBS protocol, for which the resulting UCX® have already shown promising pre-clinical outcomes in inflammatory arthritis models [9], we have selected ENZ(CG) HS protocol to be the basis of the UCX®-ATMP manufacturing.

However, before definitively committing to the ENZ(CG) HS protocol, the efficiency of the decontamination steps implemented during UCX® isolation and also throughout the UCX® expansion with regards to mycoplasma and other bacteria, fungi and endotoxins was tested. These are frequent laboratory contaminants that are clinically associated with infection, inflammation and fever in patients. We found that upon arrival, most umbilical cords contained significant bacterial and fungal contaminants, most frequently *E. coli, Streptococcus sp.* and *Staphylococcus* coagulase negative bacteria (data not shown). The initial decontamination step of the protocol was, therefore, fundamental to its success. For the final protocol, only material and reagents that were endotoxin-free were used throughout the isolation and cell expansion. Quality control points were set at various passages and detection assays performed to each corresponding sample. While 75% and 100% of the cultures of UCX® derived from ENZ(RG) FBS protocol were endotoxin positive at P0 and P1, respectively (n = 6); no contamination was detected in UCX®-ATMP, derived from ENZ(CG) HS, until at least P20 (cultures tested every five passages; n = 20 - sensitivity 0.12 EU/mL). Furthermore, since no bacterial or fungal contaminations were detected in UCX®-ATMP samples derived from the ENZ(CG) HS protocol, we considered the decontamination step applied to be sufficient to ensure a contaminant-free UCX® product. Therefore, cells from P0 onwards were expanded in antibiotic/antimycotic free media, an added measure to comply with more strict local authority requirements for a given ATMP to reach the clinic. These results confirm that the efforts in material and reagent selection performed were successful in the prevention of microbial, mycoplasma and endotoxin contamination of the final UCX® product.

Based on accumulated evidence related to cell yields, phenotypes, transcriptome profiles and level of microbiologic and endotoxin contaminations, we have finally selected the ENZ(CG) HS protocol to be the basis of the UCX®-ATMP manufacturing, and thus subjected the resulting cells to further characterization.

### Further characterization of UCX®-ATMP derived from the ENZ(CG) HS protocol

#### Quality data

Similar to UCX®, the isolation of UCX®-ATMP is a robust and efficient protocol; a 97% success rate for isolation and expansion was obtained from a statistically relevant UC sample population (n = 28).

UC samples have an intrinsic variability that we do not control. The composition of each cord varies according to time of gestation, mother’s genetic inheritance, sample manipulation and storage post-partum and so on. These differences cause an intrinsic variation in the number of primary cells isolated from the tissue. Notwithstanding, cell isolation is almost always guaranteed, with an average of 1.63 × 10^5^ cells/g obtained immediately following UC digestion (and before cell adherence to the plastic surface). Cell viability at this stage averages 93%. This population is extremely heterogeneous, with a MSC surface marker profile far from meeting the minimum ISCT criteria (Additional file 2: Table S1). However, after plastic adherence, which remains one of the most important steps to isolate MSCs [2], a more pure cell population is obtained, in full agreement with the ISCT MSC criteria over various passages (up to P20) (see below). Furthermore, cells were regularly seeded and cell viability determined at each seeding point by trypan blue exclusion. Viability after detachment was systematically >90%. Hence, at the end of P0, UCX®-ATMP is a relatively homogeneous MSC cell population.

**Figure 5:**
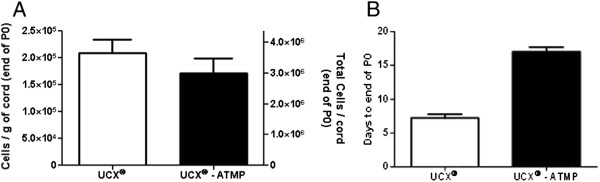
**Isolation of UCX® and UCX®-ATMP. (A)** Comparison of the cell yield in number of cells per gram (g) of UC (left Y-axis) and total cells obtained per UC (right Y-axis) and **(B)** days required to reach confluency (end of P0). Results are represented as mean ± SEM.

With this method, an average of 3 × 10^6^ UCX®-ATMP cells (about 170,000 cells/g cord), was obtained at the end of passage 0, within an average time of 17 days (Figure 5).

**Figure 6:**
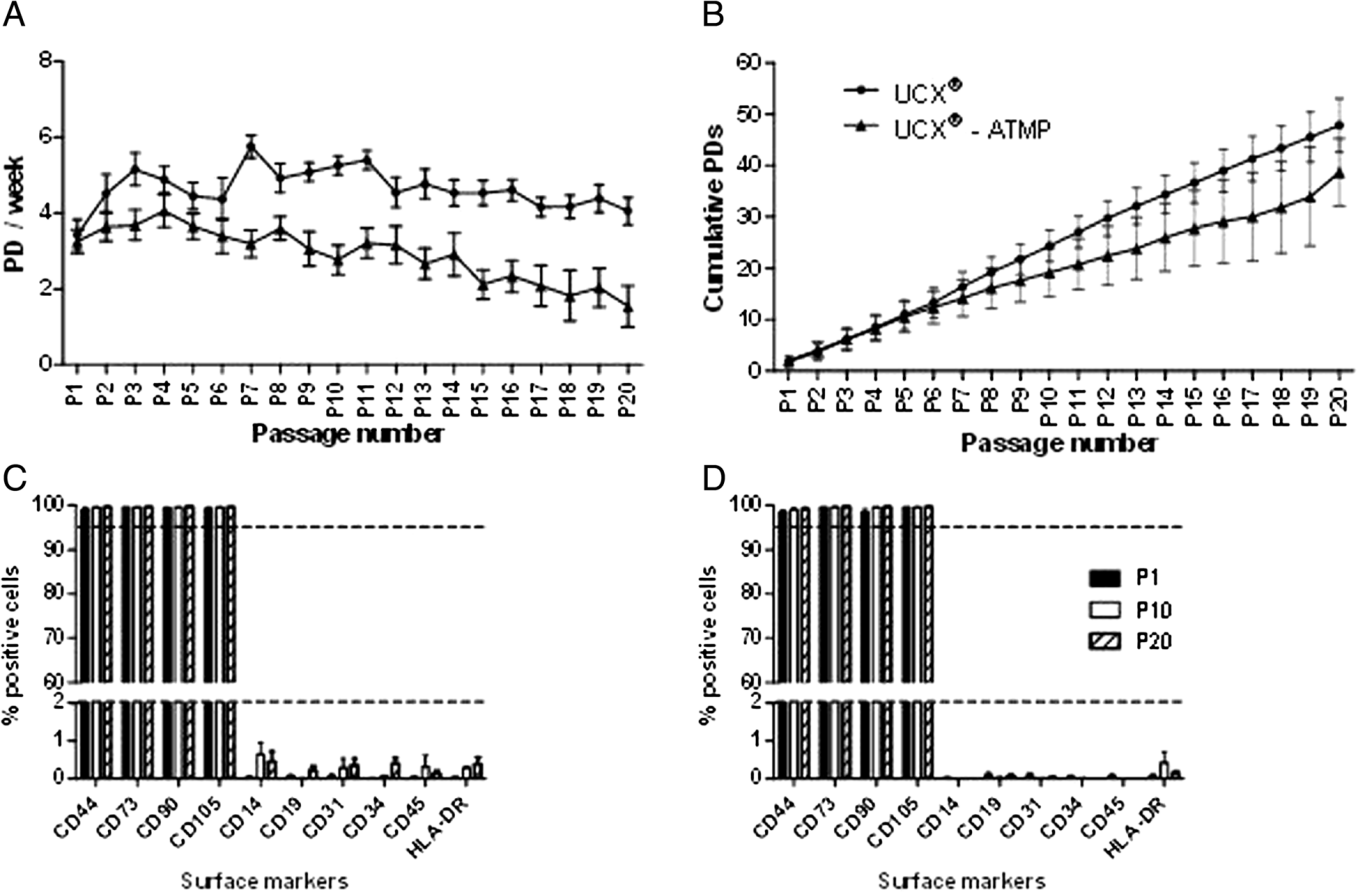
**UCX® and UCX®-ATMP characterization. (A)** Population doubling rate and **(B)** cumulative population doublings of cells from passages 1 to 20. UCX®-ATMP propagated well, with growth rates ranging between 1.5 to 3.5 population doublings (PDs)/week presenting a considerable longevity in culture, allowing for very high cell expansion potential. The comparative control UCX®, grew at 3 - to 6 PDs/week until P20 and reached a maximum passage of P25 after 54 ± 10 PDs and 112 ± 21 days in culture. Flow cytometric analysis of ISCT defined mesenchymal stromal cell (MSC) markers of UCX® **(A)** and UCX®-ATMP **(B)** at passages 1, 10 and 20. The percentage of CD44 and CD31, common MSCs markers, was also determined. Results are represented as mean ± SEM.

Comparison of UCX® and UCX®-ATMP was performed at later passages with respect to cell expansion potential and stability. Results showed that cells grow at a steady rate. Although lagging with respect to UCX®, UCX®-ATMP propagated well, with growth rates ranging from 1.5 to 3.5 population doublings (PDs)/week presenting a considerable longevity in culture, allowing for very high cell expansion potential (an average of passage 18, after 30 ± 10 PDs and 96 ± 25 days in culture) (Figure 6A, B).

**Figure 7:**
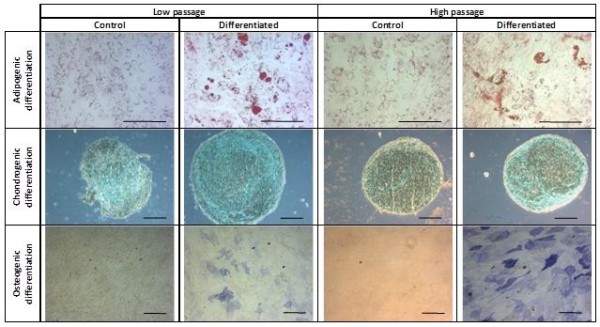
**Differentiation potential of UCX®-ATMP.** Cells underwent differentiation at low and high passages as detected by the respective biochemical staining; Oil red O staining for adipogenic (bar = 100 μm), Alcian Blue staining for chondrogenic and Alkaline Phosphatase activity staining for osteogenic differentiation (bar = 200 μm).

Furthermore, the MSC surface marker expression profile of UCX®-ATMP was compliant with ISCT guidelines for at least 20 passages (Figure 6C, D) and their differentiation potential was maintained to at least P15 (Figure 7). The fact that UCX®-ATMP cells can undergo considerable expansion, maintaining their expected MSC phenotype, including tri-lineage differentiation potential, is of great importance considering the high cell number required for their use in a clinical setting which often implies the establishment of master and working cell banks.

#### Safety data

Together with efficacy, the absence of risk for the recipient’s safety is of most importance for ATMP certification and market authorization for a cellular product. In this study we addressed genetic stability, potential for teratoma formation, as well as possible effects upon allogeneic administration (immune response).

**Figure 8:**
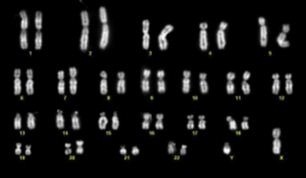
**G-banding analysis of UCX®-ATMP at P7.** Normal karyotype 2n = 23, autosomal and sex chromosomes.

To confirm the genetic stability of UCX®-ATMP throughout the expansion process, cells were analyzed at the cytogenetic level at various passages. Analysis (20 metaphases) confirmed that no significant chromosomal aberrations were found up to P15 (Figure 8). Furthermore, UCX®-ATMP cells implanted beneath the testicular capsule of young SCID/Beige mice confirmed the lack of teratoma-forming potential of these cells for at least 8.5 weeks. While UCX®-ATMP cell-implanted animals were 100% negative for teratoma formation, positive control human embryonic stem cells (H-9) induced teratomas in 80% of the control group test animals (data not shown).

**Figure 9:**
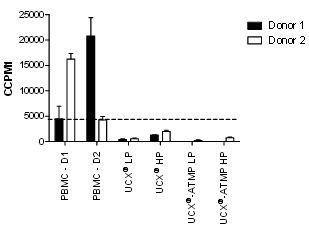
**Allogeneic lymphocyte stimulation reaction of UCX® and UCX®-ATMP.** Peripheral blood mononucleated cells (PBMCs) of donor 1 (filled bars) and donor 2 (open bars) were co-cultivated with PMBCs, UCX® and UCX®-ATMP at high (P15) and low (P5) passage (HP and LP). Proliferation was measured through a [3H]thymidine incorporation assay and results are expressed in counts per minute (CPM). Basal proliferation level is marked at approximately 5,000 CPM. Results are represented as mean ± SEM.

**Figure 10:**
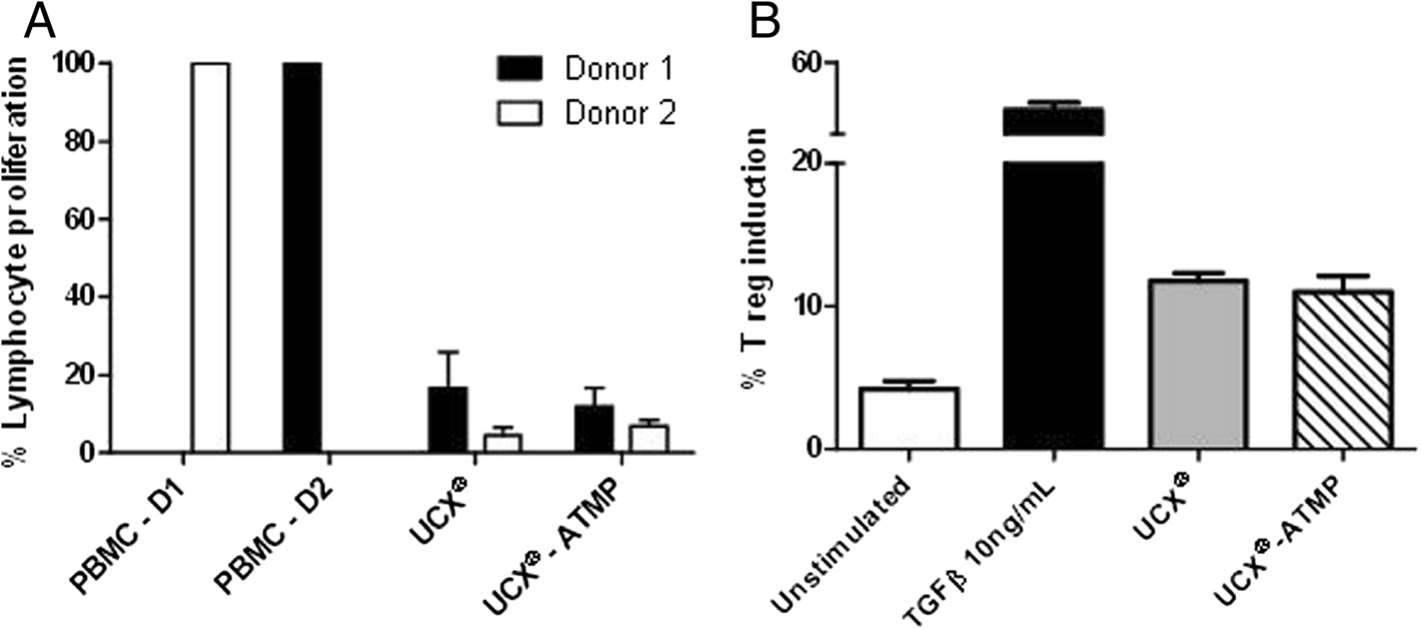
**Immunosuppression of UCX® and UCX®-ATMP. (A)** Suppression of T cell activation was assayed by incubating CD3, CD28 and IL2 activated peripheral blood mononucleated cells (PBMCs) from two different donors with irradiated UCX® and UCX®- advanced therapy medicinal product (ATMP) and measuring lymphocyte proliferation **(B)** Treg conversion was measured in the same cells by culturing irradiated cells with CD3^+^CD4^+^CD25^-^ T-cells and comparing the percentage of converted CD3^+^CD4^+^CD25^+^Foxp3^+^ regulatory T-cells with unstimulated cells (negative control) or induced conversion with TGFβ. Results are represented as mean ± SEM.

Lymphocyte proliferation assay measures the ability of lymphocytes to undergo proliferation when stimulated *in vitro* by a foreign molecule, antigen or mitogen. Immunogenicity of UCX®-ATMP was measured by the level of proliferation of PBMCs in co-culture. Mixed lymphocyte stimulation reactions were performed with two different donors in order to reduce the probability of HLA matching between responders (PBMCs) and stimulators (UCX®-ATMP) (Figure 9). The effect of high and low passage UCX®-ATMP was assessed and lymphocyte proliferation measured. UCX® derived from the ENZ(RG) FBS protocol were also included as a comparative control [9]. Results showed that UCX®-ATMP did not elicit lymphocyte activation at any level, indicating low immunogenicity. Furthermore, basal proliferation of lymphocytes was reduced when in culture with UCX®-ATMP, suggesting an UCX®-ATMP-mediated an immunosuppressive effect in the culture environment. This was confirmed with immunosuppression assays by using either co-cultured UCX® (as a comparative control) or UCX®-ATMP with CD3, CD28 and IL2 activated PBMCs, again from two different donors, and measuring lymphocyte proliferation (Figure 10A). Results clearly showed that just like UCX®, UCX®-ATMP are immunosuppressive. This immunoregulatory property was also observed in a Treg (CD3^+^CD4^+^CD25^+^Foxp3^+^) conversion assay, where the presence of UCX®-ATMP resulted in a significant conversion of the CD4^+^ cells to CD4^+^CD25^+^Foxp3^+^Tregs (Figure 10B). Together, these results confirmed that UCX®-ATMP are not only non-immunogenic, an essential characteristic for future allogeneic application in cell therapies, but are also capable of suppressing the immune system by 1) inhibiting T cell proliferation and 2) inducing Tregs.

### Cryopreservation and recovery of UCX®-ATMP

The isolation method herein described allows for the harvesting of a substantial amount of UCX®-ATMP that are easily expanded in culture, reaching clinically significant cell numbers. However, successful long-term cryopreservation is fundamental for proper recovery prior to future applications. For the UCX®-ATMP, the aim was to maintain a FBS-free cellular product and remove potentially hazardous substances such as DMSO. We, therefore, implemented the use of Biofreeze®, an animal component-free, DMSO-free cryopreservation solution. A controlled rate freezer (CRF) was used and the most optimal cryopreservation profile was selected (comparative results not shown). The CRF protocol adapted from Freimark *et al*., 2011, along with the use of Biofreeze, was selected since the resulting viable cell recovery rate was 98%, comparable to the research grade cryopreservation using FBS and DMSO. Thawed UCX®-ATMP adhered to the plastic surface of the culture flasks and maintained a cell morphology similar to cells prior to cryopreservation. Furthermore, cells grew to confluence (within three to five days) and maintained a typical growth rate of 3.0 PDs/week. Additionally, post-thawed UCX®-ATMP maintained the MSC phenotype regarding cell surface markers and differentiation potential (data not shown).

## Discussion

The increasing number in clinical trials involving stem cell therapies over the last few years is helping to shape the regulatory demands for emergent new medicines. However, there is still a lack of definitive standards for the production of clinical-grade cell products. In fact, according to EMA’s Directive 2001/83/EC applied to Advanced Therapy Medicinal Products (EMA/CAT/CPWP/686637/2011 - Committee for Advanced Therapies (CAT)), the concept behind ATMP development should not be based on fixed pre-established criteria. Instead, and due to the diversity and complexity of cell based biopharmaceuticals, the strategy should be further adaptable to a case-by-case basis, always keeping in mind a strict focus on risk assessment towards safety to the end user.

In the case of UCX®, adaptation to ATMP-compliant conditions started with an effective decontamination procedure of the tissue source by using an antibiotic and antimycotic solution. This allowed for the removal of these agents from the subsequent isolation and cell expansion steps. GMP-compatible and clinical-grade materials and reagents were introduced. Endotoxin and mycoplasma free materials were used throughout the isolation and culture procedures. More specifically, a research grade, crude collagenase, known to contain endotoxins, non-specific peptidases and xenoproteins [20], was replaced with a defined, clinical grade enzyme, without compromising cell yield. Trypsin, an animal derived enzyme, was also replaced by animal origin-free TrypLE Select. Though FBS is currently used in cell cultures in ongoing clinical trials, its use is controversial. EMA has particular concerns regarding the use of FBS due to the widespread presence of bovine spongiform encephalopathy and the potential contamination of samples. Also, growing evidence shows that the presentation of FBS proteins, such as albumin, to the recipient immune system may result in subsequent antibody-based responses with the risk of serum sickness [21]. Therefore, in the likelihood that FBS be excluded for use in clinical products, and foreseeing a future clinical application of UCX®, HS and hPL substitutes were introduced and tested in both the initial isolation steps and cell expansion. Results showed that both substitutes affected yield. In BM-MSCs, hPL has been shown to enhance proliferation when compared to FBS [22-24]. In growing cultures of UCX® cells, hPL had a similar effect (Additional file 1: Figure S1) but isolation efficiency was seriously compromised when hPL was used immediately following UC digestion. Despite inducing an up-regulation of genes related to adhesion (and initial up-regulation of CD31 protein expression, see Figure 4), hPL seems to mainly affect cell adhesion upon UC digestion, which ultimately affects yield. While in hPL-supplemented cultures, reduced cell isolation efficiency was associated with poor initial cell adherence, in HS supplemented cultures, it was associated with slower proliferation. Indeed, the proliferation rate of UCX®-ATMP cells was slightly lower than UCX® (Figure 6A). The latter was consistent with the DAVID-Functional Annotation Clustering analysis showing that the down-regulated genes in HS cultures were mostly enriched for matches within the “cell cycle, regulation, transcription” functional annotation group.

Despite lower proliferation after cell isolation, HS allows for efficient cell isolation, comparable to that of our research-grade protocol. The use of HS is associated with the humanization of the culture, a clear advantage over the use of FBS, and a viable option for the isolation and initial culture of UCX® in xenofree conditions. Interestingly, it has also been suggested that HS maintains MSCs in a more primitive stage [25]. Issues concerning variability and limited availability, often associated with the use of HS, can be overcome by the use of large, pre-tested, donor pools that can be better adjusted for a predefined quality as an “off the shelf” product. Most commonly, serum from AB donors are used to avoid the presence of isoagglutinins, more as a precaution, since MSCs do not appear to express ABO blood group antigens [26]. For the pathogen screening of the serum, quarantine storage can in part overcome the risk of the diagnostic window. This will allow a retest of the blood donor after a second donation, and after a time interval of at least four months. Only those units derived from donors verified, after the second donation, to be negative for human infectious disease markers can then be pooled and used [27]. Various pathogen-reduction strategies to treat blood products are also under investigation [28].

FBS substitution for HS resulted in a down-regulation of important immune-related genes, such as *HGF*, *IGFBP5*, *IL1B* and *IL6* (Table 1). In addition, expansion of MSCs could compromise immune properties [29] and could potentially induce genomic aberrations. Nonetheless, much like UCX®, UCX®-ATMP showed low immunogenicity (Figure 9) and were found to be immunosuppressive (Figure 10), despite passage number (data not shown). Further, UCX®-ATMP presented no apparent safety concerns upon expansion in that no genomic aberrations were detected up to passage 15 (Figure 8) and no tumorigenicity (teratoma formation) potential was exhibited. The latter is not surprising in light of recent data showing that though MSCs are recruited to tumor sites, no increase in leukemia relapse has been reported after MSC-treatment in graft-versus-host disease (GvHD) [30]. Also, since the first clinical trial in humans using MSCs in 1995, tumors have never been reported [2].

Finally, long-term storage of cells is critical to ensure a reliable supply for health care providers. The majority of published work is based on cryopreservation of cells in DMSO as a cryoprotectant agent, often in combination with FBS. Although DMSO is used routinely, it has potential adverse effects on the recipient and may not be optimal for all cells [31]. Furthermore, FBS is also associated with potential contaminations and immune response risks as previously noted. In addition, most academic laboratories store cryopreserved cells submerged in liquid nitrogen, which is not a feasible option for GMP-grade cell products due to high risk of cross-contamination [32]. Instead, GMP guidelines demand that cells must be maintained in liquid nitrogen vapor-phase individual containers. Taking into consideration these safety and storage factors, cryopreservation of UCX®-ATMP was optimized using a therapy-grade, animal component-free and DMSO-free cryopreservation product, allowing for GMP-adaptable conditions for UCX®-ATMP cryopreservation in N_2_ vapor phase. Most importantly, thawed UCX®-ATMP maintain ISCT guideline criteria for MSCs, as well as the associated safety aspects and, therefore, these conditions can be used for the establishment of a UCX®-ATMP cell bank.

## Conclusions

Overall, our process upgrade results demonstrate the stability and safety of UCX®-ATMP as a cell product and support the use of these cells as an ATMP active substance. For a prospective clinical use of UCX®-ATMP, further potency assays will be used with a given application in mind. Ideally, these tests should be standardized and validated quantitative tests as it has been proposed for bone marrow-derived and adipose tissue-derived MSCs [33].

## Abbreviations

ATMP: Advanced therapy medicinal product; CRF: Control rate freezer; DMSO: Dimethyl sulfoxide; EMA: European Medicines Agency; ENZ(CG): Enzyme (Clinical Grade); ENZ(RG): Enzyme (Research Grade); FBS: Fetal bovine serum; FDA: Food and Drug Administration; GMP: Good manufacturing practices; hPL: Human platelet lysate; HS: Human serum; ISCT: International Society for Cellular Therapy; MSCs: Mesenchymal stromal cells; PBMCs: Peripheral blood mononuclear cells; PD: Population doubling; UCX ®: Human umbilical cord tissue-derived mesenchymal stromal cells.

## Competing interests

PEC and HJC are shareholders and JPM, RNB, JA, MF, MT and JMS are currently employed at ECBio S.A., a company developing cellular therapeutics based on human mesenchymal stromal cells. All authors declare not to have any competing interests beyond the scope of the work presented.

## Authors’ contributions

JPM, participated in collection of data on cell isolation, culture and preservation, data analysis and interpretation, conception and design, manuscript writing and revision. JMS contributed to conception and design, manuscript writing and revision. JA, MF and MT participated in collection of data on cell isolation, culture and preservation, data analysis and interpretation, and manuscript revision. SA, AAD and AV took part in the collection of data on cell immunology, data analysis and interpretation. MG, BS and KEJD were involved in the collection of tumorigenicity and karyotype data, data analysis and interpretation. SP participated in collection of genetic expression data, and data analysis and interpretation. WL, LG and RNB took part in data analysis and interpretation, conception and design, manuscript writing and revision. EA participated in conception and design, and manuscript revision. PE and HC were involved in obtaining financial support, and in conception and design, and manuscript revision. All authors read and approved the final manuscript.

## Supplementary Material

Additional file 1: Figure S1 Cumulative population doublings of UCX® from three different cords isolated with FBS containing media. Click here for file

Additional file 2: Table S1 Flow cytometric analysis of cell surface markers in UCX®-ATMP immediately after isolation. Click here for file
