# Supplementary figures and images for: Towards an advanced therapy medicinal product based on mesenchymal stromal cells isolated from the umbilical cord tissue: quality and safety data
Source: Stem Cell Res Ther. 2014 Jan 17;5(1):9. doi: 10.1186/scrt398 (PMC4055140; doi:10.1186/scrt398)

Additional file 1: Figure S1


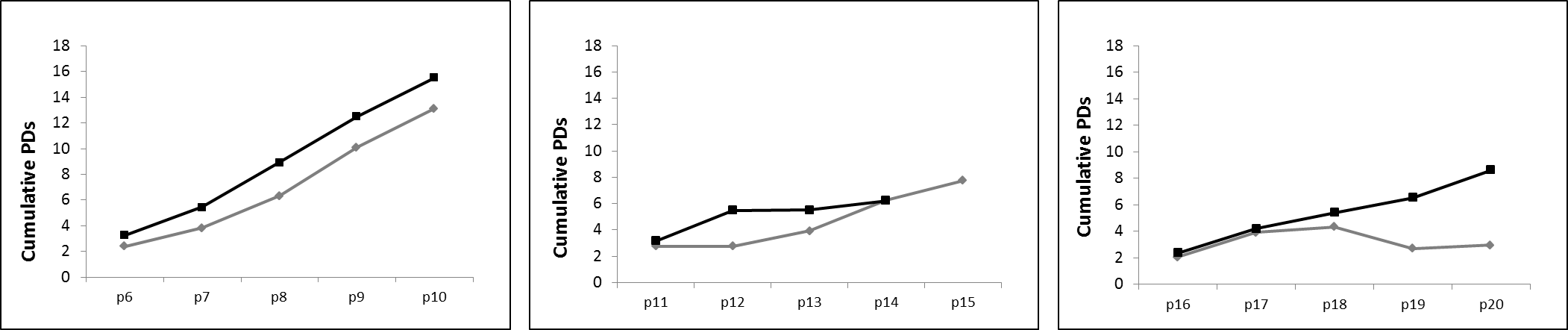

Supplement: Additional file 1: Figure S1 — Cumulative population doublings of UCX® from three different cords isolated with FBS containing media. [file scrt398-S1.docx]
