# Supplementary material for: Towards an advanced therapy medicinal product based on mesenchymal stromal cells isolated from the umbilical cord tissue: quality and safety data
Source: Stem Cell Res Ther. 2014 Jan 17;5(1):9. doi: 10.1186/scrt398 (PMC4055140; doi:10.1186/scrt398)
Supplement: Additional file 2: Table S1 — Flow cytometric analysis of cell surface markers in UCX®-ATMP immediately after isolation. [file scrt398-S2.docx]

Additional file 2: Table S1

| **MSC Markers directly after digestion of UC** | % positive cells | SD |
| --- | --- | --- |
| **CD73** | 45 | 0,0 |
| **CD105** | 22 | 1,1 |
| **CD90** | 85 | 7,6 |
| **CD44** | 16 | 5,3 |
| **CD14** | 4 | 0,4 |
| **CD45** | 5 | 0,8 |
| **CD34** | 0 | 0,2 |
| **CD31** | 3 | 0,4 |
| **CD19** | 0 | 0,0 |
| **HLA-DR** | 3 | 3,8 |
